# A non-destructive, fast, inexpensive, non-toxic chelating resin-based DNA extraction protocol for insect voucher specimens and associated microbiomes

**DOI:** 10.1093/jisesa/ieaf062

**Published:** 2025-06-03

**Authors:** Morgan E Brown, Sara Ottati, Valeria Trivellone

**Affiliations:** Illinois Natural History Survey, Prairie Research Institute, University of Illinois Urbana-Champaign, Urbana-Champaign, IL, USA; Department of Entomology, University of Illinois Urbana-Champaign, Urbana-Champaign, IL, USA; Institute for Sustainable Plant Protection, National Research Council of Italy, Turin, Italy; Department of Plant Biology, Uppsala BioCenter, Swedish University of Agricultural Sciences and Linnean Centre for Plant Biology, Uppsala, Sweden; Illinois Natural History Survey, Prairie Research Institute, University of Illinois Urbana-Champaign, Urbana-Champaign, IL, USA

**Keywords:** vouchering, Auchenorrhyncha, phytoplasma, specimen curation, nucleic acids

## Abstract

Identifying a DNA extraction method that yields high quantity and quality DNA is a crucial component of molecular ecological studies; and the best suited method can vary greatly depending on research priorities. Here, we propose a nondestructive extraction method for insect museum vouchers aimed at analyzing gut-associated microbiomes. The leafhopper *Euscelidius variegatus* (Kirschbaum) (Hemiptera: Cicadellidae) associated with the bacterial plant pathogen Flavescence dorée phytoplasma, a member of the genus ‘Candidatus *Phytoplasma*’ (Mollicutes: Acholeplasmataceae), was used as an experimental model. We developed and refined a resin-based DNA extraction protocol by testing the effects of prelysis bleaching and postlysis proteinase K inactivation on DNA quality and yield. We found that bleaching did not compromise the integrity of insect and associated bacterial DNA and that excluding the inactivation of proteinase K did not interfere with quantitative polymerase chain reaction analysis. Based on our findings, we recommend a DNA extraction protocol for insect voucher specimens and associated microbiomes that includes a prelysis bleaching step to chemically degrade external contaminants without proteinase K inactivation, thereby reducing processing time. Our refined protocol resulted in a high DNA yield, which we successfully analyzed using quantitative polymerase chain reaction analysis and other downstream molecular applications, including targeted high-throughput sequencing.

## Introduction

DNA extraction is a critical step in molecular ecological studies, and the selection of an effective extraction method is crucial to obtain high-quality DNA for downstream applications, such as quantitative polymerase chain reaction (qPCR) amplification and sequencing. Numerous methods for the extraction of nucleic acids have been proposed to accommodate a variety of sample types (Dairawan and Shetty 2020), tailored to specific research priorities, including target organism, research goal, cost, and operator safety concerns (Shin 2013).

Detection of microbial associates in invertebrate hosts relies on efficient DNA extraction performed on the whole host body (Petersen and Osvatic 2018, Andriienko et al. 2024). Recently, the use of insect vouchers preserved in museum collections has gathered attention due to their potential use in unveiling elusive microbe-host associations, particularly when studying insect-borne parasites (DiEuliis et al. 2016). Museum collections have become an increasingly important resource for understanding parasite diversity, ecology, and evolution. They allow researchers to track past host–pathogen associations, document associations in collection sites that are no longer accessible, revise the taxonomic status of the associates, and resample the associations over time as new technologies become available (Dunnum et al. 2017, Colella et al. 2021, Trivellone et al. 2021, Nelder et al. 2024).

Prior studies focused on soft-bodied small insects have compared the efficacy of different extraction methods, many of which guarantee homogeneous cellular digestion prior to DNA purification by crushing the whole or part of the insect body, thereby maximizing DNA yield (Junqueira et al. 2002, Asghar et al. 2015, Jangra and Ghosh 2022). However, methods that rely on crushing are not suitable for long-lasting preservation of museum specimens. Several methods have been developed that successfully extract and purify DNA while leaving the insect exoskeleton intact (Favret 2005, Pons 2006, Gilbert et al. 2007, Rowley et al. 2007, Hunter et al. 2008, Castalanelli et al. 2010, Carew et al. 2018, Cilia et al. 2022, Andriienko et al. 2024). However, many of these methods utilize hazardous chemicals, such as phenols or chloroform (Lienhard and Schäffer 2019, Guo et al. 2022). On the other hand, nontoxic protocols available as commercial kits may be costly, require specialized equipment, or be unsuited for work on insects (Smith et al. 2003, Schiebelhut et al. 2017).

Resin-based DNA isolation methods utilize negatively charged surfaces to purify nucleic acids by binding metal ions and positively charged proteins. These methods, such as those using chelating resin, are known to be nontoxic, cost-effective, and suitable for downstream application (Asghar et al. 2015, Miura et al. 2017, Lienhard and Schäffer 2019, Pacheco et al. 2023, Hackett et al. 2024). Nevertheless, such protocols often employ a destructive approach which involves grinding or crushing the specimen’s body to improve penetration of cells containing nucleic acids. This approach is not suitable for museum specimens, which may be rare and irreplaceable, and for groups where taxonomic uncertainty requires vouchering to document the identity of the species under study. Instead, nondestructive methods can be implemented to keep the specimen intact. However, unlike destructive approaches, nondestructive methods may not ensure homogeneous digestion of tissues, potentially affecting not only the final yield and quality of extracted DNA, but also the quantification of associated low-copy microorganisms.

Moreover, nondestructive methods often rely on proteinase K (PK), a broad-spectrum serine proteinase, to digest cellular proteins and facilitate cell lysis during DNA extraction. PK is widely used for nucleic acid extraction and is sometimes inactivated with heat after extraction to prevent continued enzymatic activity, which could lead to further unintended lysis and potential negative impacts on subsequent analyses. Burkhart et al. (2002) found that the use of PK in DNA digestion caused inhibition of PCR in mouse tail DNA samples, raising concern that this interference may occur in other DNA samples intended for downstream analysis. However, Lienhard and Schäffer (2019), employing a destructive extraction method, found that DNA yield was greater in samples that did not undergo PK inactivation compared to samples that did undergo heat inactivation of PK. Furthermore, they did not report any disturbance in PCR analyses as a consequence of residual PK activity. Moreover, the effects of PK inactivation on DNA yield under a nondestructive method, particularly for the quantification of insect-associated microorganisms, remain unknown.

One important consideration when extracting DNA from insect samples for microbiome analyses is the potential presence of contaminants containing environmental DNA from various sources on external surfaces of the insect’s body (Jüds et al. 2023). Greenstone et al. (2012) demonstrated that bleaching insect bodies prior to DNA extraction effectively eliminated potential sources of DNA contamination. Huszarik et al. (2023) further confirmed that bleaching reduces external contaminants and showed that DNA extracted from bleach-treated specimens could still be successfully amplified via PCR and sequenced via DNA metabarcoding. While specimens treated with bleach as described in these studies have been used successfully for analyzing insect gut content and characterizing associated microbiomes, the direct impact of bleaching on final DNA yield has not been extensively tested directly (Meyer and Hoy 2008, Moreau 2014, Arora et al. 2018, Huszarik et al. 2023).

In this paper we aimed to refine a protocol for the nondestructive extraction of nucleic acids from small insects preserved in museum collections, while providing a nontoxic, fast, inexpensive, and adequate DNA extraction method suitable for microbiome analysis. To achieve this, we investigated the effects of prelysis bleaching and postlysis PK inactivation on DNA extractions using a resin-based method. Our study targeted phytoplasmas (Mollicutes: Acholeplasmataceae), a group of wall-less bacterial plant pathogens that are harbored in the gut of moderately soft-bodied, phytophagous insects—leafhoppers (Hemiptera: Cicadellidae)—which serve as their vectors (Weintraub et al. 2019).

## Materials and Methods

### Insect Samples and Associated Bacterial Pathogens

We used adults of the leafhopper *Euscelidius variegatus* (Kirschbaum) maintained in colonies at the Institute for Sustainable Plant Protection, National Research Council of Italy (IPSP-NRC) laboratory (Turin, Italy). *E*. *variegatus* specimens used in our study ranged in length from approximately 4 to 5 mm.

This species is an efficient vector of the phytoplasma strains in the 16SrV phylogenetic group, subgroups C and D (hereafter FDp), under laboratory conditions. Healthy colonies of *E. variegatus* were reared on oat (*Avena sativa* L.) and used as phytoplasma-free insect samples in our experiments (Abbà et al. 2017, Rossi et al. 2020). For the infected insects, the phytoplasma infection rate in *E. variegatus* was maximized by exposing the insects to phytoplasma-infected broad beans for 7 d (acquisition access period), followed by 28 d on healthy broad beans (latency period). After completing the phytoplasma infection procedure, the putatively infected insects were preserved in 95% ethanol at −20 °C.

### Experimental Design

Due to a lack of consensus in the literature regarding optimal bleach concentration and exposure time for DNA decontamination, we selected a 2.5% NaOCl solution applied for 5 min as a compromise between decontamination efficacy and DNA preservation. This decision was informed by previous studies employing bleach concentrations from 0.5% to 6% with variable durations (Korlević and Meyer 2019, Koehn et al. 2019, Oh et al. 2020, de Silva Wijeyeratne and Gweon 2025). Additionally, given the concerns in the literature regarding the potential impact of residual PK activity on downstream applications, we tested whether PK inactivation might influence qPCR performance or DNA integrity in our selected associated microbes.

**Fig. 1.**
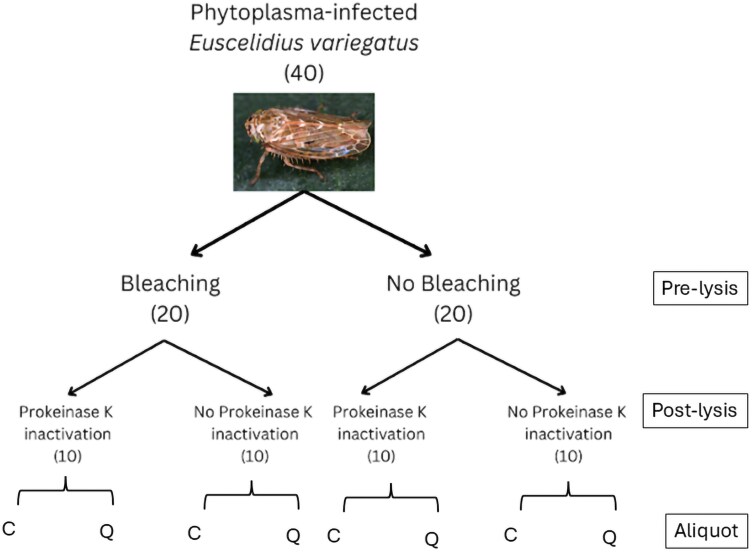
Randomization scheme for refining a resin-based DNA extraction protocol targeting microbiome and gutccontent of moderately soft-bodied museum preserved insect specimens. Phytoplasma-infected Euscelidius variegatus used for the experiments were provided by IPSP-CNR laboratory (Turin, Italy). A total of 40 individuals were randomly assigned to 4 treatments, ie each combination of bleaching (yes or no) and PK inactivation (yes or no) in pre- and postlysis, respectively. Each lysate was aliquoted to be processed with 2 DNA extraction protocols: resin-based (Chelex, C) and silica column-based (Qiagen, Q). Number of individuals per treatment is indicated in parenthesis.

The experiment uses a 2 × 2 factorial design to test the effects of prelysis bleaching and postlysis PK inactivation on the final DNA yield and quantification of host-associated microorganisms (via qPCR). A total of 40 phytoplasma-infected *E*. *variegatus* individuals (20 females and 20 males) were randomly assigned to 4 treatments: (i) bleaching + no PK inactivation; (ii) no bleaching + no PK inactivation; (3) bleaching + PK inactivation; and (4) no bleaching + PK inactivation. Each treatment included 10 biological replicates and 1 phytoplasma-free individual used as a negative control for phytoplasma quantification. After the lysis step, each lysate sample was divided into 2 equal portions (paired samples) to complete the DNA extraction. One portion underwent a resin-based extraction method, while the other was processed using a commercial silica column-based method, which served as a reference for high-quality DNA recovery. A total of 80 paired samples were processed (Fig. 1).

### Preparation of Samples and Cell Lysis

A total of 40 phytoplasma-infected *E. variegatus* individuals were placed in single vials containing 95% ethanol (EtOH) to preserve DNA prior to extractions (King and Porter 2004, Nagy 2010). For specimens treated with bleach, each individual was immersed in a 2.5% bleach solution (NaOCl—The Clorox Co., Oakland, CA, USA) for 5 min to eliminate or degrade potential contaminants from external body surfaces. To evaluate potential impacts on DNA integrity, we included appropriate controls in subsequent qPCR assays.

Cell lysis was performed using TES buffer (20 mM Tris, 10 mM EDTA, 0.5% SDS, pH ~ 7.0) and PK. Each insect body was placed in a microcentrifuge tube containing 50 μl TES buffer and 2 μl PK solution 20 mg/ml (Invitrogen, catalog number 4333793), then incubated in a 55 °C water bath for 24 h to ensure thorough protein and enzyme digestion. Following incubation, the insect bodies were removed from the tubes and preserved in 95% EtOH for later examination and vouchering. The extracted DNA samples were either subjected to PK inactivation by incubating at 95 °C for 10 min or left without inactivation. Each sample was aliquoted (see Experimental Design) and assigned to one of the selected DNA purification strategies, chelating resin, or a commercial silica column-based kit.

### Vouchering and Photographing

Extracted specimens were point-mounted, labeled, and deposited in the Illinois Natural History Survey Insect Collection (INHS Insect Collection codes 1071801-1071844). Both extracted and unextracted specimens from all combinations of extraction treatments (1—bleaching + no PK inactivation; 2—no bleaching + no PK inactivation; 3—bleaching + PK inactivation; and 4—no bleaching + PK) were photographed to assess changes in specimen coloration and condition that may have occurred during the extraction process. A photograph of a museum specimen collected in 1968 without the use of EtOH was also photographed to assess possible impacts of EtOH as a preservation method. Dorsal and ventral images of whole-body specimens were taken using a Canon DX1 SLR camera (Canon Inc., Tokyo, Japan) equipped with a Canon MP-E 65mm macro lens mounted to a motorized lift. Images were captured at multiple focal planes and stacked using Zerene Stacker software (Zerene Systems, Richland, WA, USA).

Images of genitalia were taken from one individual that had not undergone extraction and another that underwent the most intensive extraction treatment (bleaching + PK inactivation), to assess potential alteration to diagnostic characters, including the aedeagus and subgenital plates. Images were captured using a Jenoptik Gryphax Arktur microscope camera (Jenoptik Optical Systems GmbH, Jena, Germany) mounted to an Olympus BX41 microscope (Olympus Corp. Tokyo, Japan). Photographs were taken at multiple focal points and combined using Jenoptik Gryphax software (Jenoptik Optical Systems GmbH, Jena, Germany).

### Resin-based DNA Extraction

Bio-Rad Chelex 100 Resin was used for resin-based DNA extractions. Chelex was added after incubation with the lysis buffer to precipitate undesired molecules, such as Mg^2+^ ions, leaving the DNA in the supernatant. Details of the Chelex suspension and precipitation protocol can be accessed at DOI: 10.17504/protocols.io.bp2l6x54rlqe/v1.

### Silica Column-based DNA Extraction

Due to its widespread use and demonstrated efficacy in extracting DNA from insects (Mullin et al. 2023), the Qiagen DNeasy Blood & Tissue Kit was chosen as a quality reference (baseline) for evaluating the resin-based protocol tested in this study. Since the manufacturer’s protocol is not designed for nondestructive lysis, we implemented a slightly modified version to enhance comparability among treatments (see Supplementary Table S1). Specifically, we substituted the Qiagen ATL buffer with TES buffer to avoid introducing an additional variable, as our primary focus was on assessing the effects of the treatments under investigation. While we acknowledge that the use of TES buffer may have influenced the performance of the Qiagen kit, the objective of our study was not to directly compare the 2 extraction methods, but rather to evaluate the impact of bleaching and PK inactivation on the resin-based DNA extraction protocol.

### DNA Yield and Quality Evaluation

Since we targeted total DNA from insects and their associated microorganisms, specifically pathogenic bacteria that may be present in very low titers, we measured total DNA yield, integrity, purity, and microorganism quantification to ensure a comprehensive assessment.

A total of 80 DNA templates were quantified using the Qubit 3.0 Fluorometer (Invitrogen) and the Qubit dsDNA Quantification Assay Kit (Invitrogen) according to the manufacturer’s instructions. Total DNA yield (ng/sample) was calculated from the recorded concentrations. Using a NanoDrop Microvolume Spectrophotometer (Thermo Scientific), the purity of nucleic acids was evaluated by comparing the 260/280 absorbance ratio of a subset of 16 samples. If the 260/280 absorbance ratio is ~1.8 the DNA is considered optimal. Any deviation from this value indicates the presence of leftover contaminants (Wilfinger et al. 1997).

Gel electrophoresis was carried out on 10 samples that underwent the finalized protocol to estimate fragmentation. A mixture of 1% agarose in 1× TBE (tris, borate, EDTA) buffer was prepared. When possible, 100 ng of DNA was loaded into each well and electrophoresed before being photographed on a blue light box. A 1 kilobase pair ladder (New England Biolabs) was included as a reference for DNA weight.

To further evaluate the quality of the DNA obtained with our resin-based approach, particularly for the target microbe, we used a high-throughput next-generation DNA sequencing approach, Anchored hybrid enrichment (AHE). The AHE protocol was carried out as described by Trivellone et al. (2022). Anchored hybrid library preparation and sequencing were conducted at Rapid Genomics LLC (Gainesville, FL, USA) using a probe kit that targets 178 phytoplasma loci, among them 45 are widely used for classification and characterization of phytoplasmas. A total of 5 samples were sequenced, including all 4 treatment combinations extracted with the resin-based method, as well as one treatment group (no bleach and no PK inactivation) extracted using the silica-based method.

### Quantification of Phytoplasmas

To verify the effect of each treatment (bleaching and PK inactivation) on absolute quantification of bacteria (ie phytoplasmas) associated with the insect body, we used a duplex qPCR assay. To quantify phytoplasma cells, universal primers 16S-fw (5′-CGTACGCAAGTATGAAAC TTAAAGGA-3′), p16S-rv (5′-TCTTCGAATTAAACAACATGAT CCA-3′, and TaqMan probe p16S-FAM (5′-FAM-TGACGGGAC-ZEN-TCCGCACAAGCG-IBFQ-3′) targeting the 16S rDNA gene of phytoplasmas (Christensen et al. 2004) were used. Absolute quantification was achieved by normalizing the phytoplasma genome unit on the ng of total DNA. To quantify insect DNA, the 18s rDNA was chosen as the endogenous insect target; primers Au18S_1719F qFw (5′-ACTGTGTGCATGGAATAATGGA-3′), Au18S_1852R (5′-TGCGACGATCCAAGAATTTCA-3′), and TaqMan probe Au_Probe_1796-Hex (5′-AGGGACAGGCGGGGGCATTCG-HEX-3′) were used. Given the consistently low DNA concentrations yielded by insect voucher specimens, especially very small-bodied taxa (<3 mm), and our goal to evaluate qPCR performance under worst-case conditions, we deliberately used minimal DNA input to simulate field-relevant challenges. Specifically, 1 μl of DNA (0.1 ng) was used in a reaction mix of 10 μl total volume, containing 1× TaqMan Universal PCR Master Mix (Invitrogen), 160 nM of each of the 4 primers and 160 nM of each of the 2 TaqMan probes. Each sample was run in duplicate in a CFX Opus Real-Time PCR Systems (Bio-Rad). Cycling conditions were 95 °C for 2 min and 50 consecutive cycles at 95 °C for 15 s of denaturation followed by 1 min at 60°C of annealing and extension. In each qPCR plate, at least 4 serial 100-fold dilutions of pOP74 plasmid, harboring the target phytoplasma rDNA 16s genes, were included to calculate the phytoplasma load. Plasmid standard curve dilutions included in plates ranged from 10E + 7 to 10 target copy numbers per μl and were prepared taking into account that 1 fg of plasmids harboring phytoplasma gene portion contains 194 molecules. For insect housekeeping gene detection, a standard curve was prepared using serial dilutions (11, 1.1, 0.11, and 0.011 ng/μl) of total DNA from phytoplasma-free specimens of *E. variegatus* reared in the IPSP-CNR lab colony. Dilution series plasmid and total DNA were used to calculate qPCR parameters (reaction efficiency and R2). Mean phytoplasma copy numbers in amplified samples were automatically calculated by CFX Maestro Software (Bio-Rad) and used to express phytoplasma amount as phytoplasma genome unit/ng of insect DNA. Mean starting quantity (SQ), mean Cq values, and standard deviations for standards and nontemplate controls (NTCs) from the 5 qPCR run were used to evaluate the quality and consistency of the standard curve and to confirm the absence of contamination in NTCs (Supplementary Table S2).

DNA extracts were stored at −80 °C for long-term preservation, following protocols recommended in previous studies (Forsberg et al 2022, Tang et al 2022, Landor et al 2024).

### Statistical Analysis

To assess the effect of the 3 factors (bleaching, PK inactivation, and protocol type) on DNA yield and absolute quantification of phytoplasma, a linear mixed-effects model was performed using the *nlme* R-package (Pinheiro and Bates 2024). For each sample, aliquots were split between the resin-based and silica-based extraction methods, ensuring matched biological material across treatments. While the silica-based method was included in the model as a reference baseline, the primary aim was to evaluate how the resin-based protocol performed across different treatment combinations. To account for inherent variability associated with individual identity, individual specimens were included as a random factor. Model assumptions were validated by examining residuals for normality and homoscedasticity (Winter 2013). A posthoc Tukey test was performed on the linear mixed-effects model using the *emmeans* R-package (Lenth 2024) to further investigate treatment effects. Data were log-transformed for normalization. Data analysis and plotting were carried out in R version 4.4.0 (R Core Team 2024).

## Results

### Total DNA Yield

DNA yield extracted from bleached specimens (*n* = 40 samples) averaged 299.3 ng/sample (95% CI: 169.1 to 429.4) compared to nonbleached specimens (*n* = 40 samples) which averaged 417.1 ng/sample (95% CI: 232.1 to 602.1). DNA extracted from samples that underwent PK heat inactivation (*n* = 40 samples) averaged 264.2 ng/sample (95% CI: 147.8 to 380.6) while samples that did not undergo PK inactivation (*n* = 40 samples) averaged 452.2 (95% CI: 261.1 to 643.2). DNA processed using a resin-based protocol (*n* = 40 samples) yielded an average of 690.8 ng/sample (95% CI: 521.8 to 859.8) while DNA processed using a silica column-based protocol (*n* = 40 samples) averaged 25.6 ng/sample (95% CI: 14.8 to 36.4). All DNA yield data are reported in Supplementary Table S3. A linear mixed effect model considering nested effects of each variable on the DNA yield data revealed that protocol was significantly correlated with the resulting DNA yield (*P* < 0.001). No effects of PK inactivation, bleaching, nor interaction effects within the variables were identified (Table 1). A posthoc Tukey test revealed that the resin-based protocol involving both bleaching and PK inactivation yielded significantly less DNA than the same protocol with no bleaching and no PK inactivation (*P* < 0.05), as well as with bleaching but without PK inactivation (*P* < 0.05). The latter 2 treatments yielded similar amounts of DNA (see Supplementary Table S4 and Supplementary Fig. S1).

**Table 1.** Results of linear mixed effect model for DNA yield and absolute phytoplasma quantification. Significant factors with a *P* < 0.05 are in bold. ^†^ Proteinase K inactivation, ^‡^ Protocol.

DNA yield	Value	SE	*T*	*P*
Intercept	6.67	0.28	23.68	**<0.001**
Bleach (yes *vs* no)	−0.2	0.4	−0.49	0.63
PK in^†^ (yes *vs* no)	−0.33	0.4	−0.82	0.41
Prot^‡^ (Qiagen vs Chelex)	−3.76	0.36	−10.57	**<0.001**
Interaction term (Bleach * PK in)	−0.98	0.56	−1.74	0.09
Interaction term (Bleach * Prot)	0.89	0.5	1.77	0.08
Interaction term (PK in * Prot)	−0.35	0.5	−0.7	0.49
Interaction term (Bleach * PK in * Prot)	0.57	0.71	0.8	0.43
**Absolute phytoplasma quantification**				
Intercept	12.32	0.96	12.78	**<0.001**
Bleach (yes vs no)	0.52	1.36	0.38	0.71
PK in (yes vs no)	0.42	1.36	0.31	0.76
Prot (Qiagen vs Chelex)	−0.63	0.25	−2.53	**0.016**
Interaction term (Bleach * PK in)	−3.01	1.93	−1.56	0.13
Interaction term (Bleach * Prot)	0.44	0.35	1.26	0.22
Interaction term (PK in * Prot)	−0.18	0.35	-0.52	0.61
Interaction term (Bleach * PK in * Prot)	0.44	0.5	0.87	0.39

### Total DNA Quality

The average value of 260/280 absorbance ratio measured for the samples extracted with the silica column-based protocol (Qiagen) was 1.99 (SD: 0.858, 8 individuals) whereas an average of 1.12 (SD: 0.0874) from the same paired samples was obtained using the resin-based extraction protocol (Chelex). These results indicate optimal values for silica column-based extracted samples and the presence of organic contaminants in resin-based extracted samples (see Supplementary Table S5).

Gel electrophoresis of a subset of 10 samples showed that the inclusion of prelysis bleaching and exclusion of postlysis PK inactivation had no observable effects on the integrity of DNA extracted with both nondestructive DNA extraction protocols (see Supplementary Fig. S2).

### qPCR of Phytoplasmas Associated with the Tested Insect Samples and Sequencing

Bleaching and PK inactivation had no significant effects on downstream qPCR analysis, while the extraction protocol slightly influenced the outcome of qPCR analysis (*P* < 0.05). When comparing all bleached (*n* = 40 samples) and unbleached treatments (*n* = 40 samples), the average value of phytoplasma quantification, expressed as genome units (GU)/ng of insect DNA, was 6.082E + 5 (SD: 7.181E + 5) and 5.031E + 5 (SD: 7.181E + 5), respectively. Treatments with PK inactivation (*n* = 40 samples) had a mean quantification of 5.193E + 5 GU/ng insect DNA (SD: 6.782E + 5), while treatments with no PK inactivation (*n* = 40 samples) resulted in a mean quantification of 5.921E + 5 GU/ng insect DNA (SD: 7.578E + 5). Samples extracted following the resin-based protocol resulted in a mean phytoplasma quantification of 6.360E + 5 GU/ng insect DNA (SD: 8.104E + 5), while column-based extraction protocol had a mean phytoplasma quantification of 4.752E + 5 GU/ng insect DNA (SD: 6.057E + 5).

To assess the integrity of the barcode gene, 16Sr, sequenced from the 5 samples analyzed with AHE, we focused on the full-length phytoplasma sequence. Our assembly yielded full-length 16Sr phytoplasma sequences (1,531 bp) for the samples extracted with the resin-based approach and partial-length 16Sr sequences for the samples extracted with Qiagen (871 bp). The samples extracted with the resin-based approach showed 99.93% similarity to a reference strain of Flavescence dorée phytoplasma (16SrV, accession number AF176319.1).

### Vouchering

After nondestructive DNA extraction, specimens were temporarily returned to ethanol before long-term preservation as dry pinned specimens. In this group of insects, the primary diagnostic character lies in the male genital capsule (including aedeagus, connective, genital plates, styles) which is typically highly pigmented and often requires an additional clearing step in which specimens are soaked in 10% potassium hydroxide (KOH) solution for several hours. In our study, several potentially damaging treatments were applied and tested, including bleaching, TES buffer, exposure to PK at 56 °C for 24 h, and PK inactivation at 95 °C, and none caused any damage to the genital structures (see comparative photographs in Supplementary Fig. S3). The genital capsule of the specimen before DNA extraction was similar to that of the extracted specimen; the only difference was that the former retained more surrounding soft skin tissue, whereas in the latter, the tissue had been cleaned away (Supplementary Fig. S3a, b). Although body coloration is not a key diagnostic feature for this insect group, we compared the effects of the treatments on overall coloration and observed a slight fading of external pigmentation and patterning in specimens subjected to all combinations of treatments, though the changes were minimal and did not compromise external recognition. When comparing a specimen from our experiment preserved in 95% EtOH to an older museum voucher that was never preserved in EtOH, the only observable difference is some damage to the wing apex in the former, although this damage is likely unrelated to ethanol preservation (Supplementary Fig. S4).

## Discussion

Published nucleic acid extraction protocols are generally customized to address specific biological contexts and experimental goals, aiming to maximize both the quality and quantity of recovered nucleic acids. These criteria often include considerations for sample type, preservation methods, and the intended downstream applications such as qPCR, next-generation sequencing (NGS), or metabarcoding (Shin 2013, Sheershika and Ram 2024). In this study, our primary objective was to develop and refine a protocol for the extraction of a group of wall-less bacteria (phytoplasmas), which are obligate intracellular plant pathogens, from insect vouchers while ensuring suitability for subsequent analyses.

Our study demonstrates the applicability of a 2-step approach: first, bleaching specimens to eliminate or degrade external contaminants before lysis and second, omitting the postlysis step of PK inactivation through heating. Excluding PK inactivation reduces the total extraction time.

### Lysis and Prelysis Conditions on DNA Recovery and Downstream Analysis

Our results showed that not inactivating PK resulted in a slightly higher total DNA yield compared to the inactivated treatment. However, this difference was not consistently significant across all treatments and may reflect a spurious correlation. Our results are consistent with the findings of Lienhard and Schäffer (2019), who retrieved an adequate amount of DNA using a Chelex extraction protocol, regardless of whether PK was inactivated or not. Lysis is particularly crucial in nondestructive extraction methods, and our approach, using TES buffer and PK with prolonged exposure, proved suitable for enhancing lysis efficiency without compromising specimen morphology. This strategy was effective in retrieving high DNA yield and ensuring accurate downstream detection and characterization of microorganisms such as phytoplasmas, which may otherwise be underestimated if host tissues are incompletely lysed. Supporting the importance of effective lysis, Zhang et al. (2021) compared chloroform-based extraction with a commercial kit employing both chemical and enzymatic lysis to profile the gut microbiome of *Bombyx mori* using PCR and Illumina sequencing. Their results showed that the kit-based method was superior in recovering the underlying high-diversity bacterial community, a difference attributed to more efficient lysis of Gram-positive bacteria. This finding highlights that different protocols may not equally retrieve all bacterial species, particularly those with more robust or recalcitrant cell walls. While our protocol is effective for phytoplasmas, which are wall-less bacteria and therefore not affected by cell wall recalcitrance, lysis efficiency should be tested across a broader range of bacterial taxa to ensure the protocol’s applicability to diverse microbiomes.

Although some studies (Burkhart et al. 2002) suggest that PK may interfere with qPCR, we found no significant difference in phytoplasma absolute quantification when comparing treatments with and without PK inactivation, suggesting that leaving the PK active does not compromise the detection and amount of gut-associated bacterial DNA. These results are in line with a previous study by Wang et al. (2019), which targeted a recombinant adeno-associated virus through qPCR and showed that cycle threshold values were identical regardless of whether PK was inactivated. More recently, a study by Andriienko et al. (2024) targeting multiple microbial species across various insect hosts used a destructive extraction method to compare protocols with and without an additional alkaline buffer step at high temperature (HotSHOT: 20 to 18 min at 65 °C followed by 2 min at 98 °C) prior to lysis with “Vesterinen” lysis buffer and PK. Their findings showed that the HotSHOT step led to decreased microbial quantification, although it had minimal impact on the composition of abundant microbial species. This decrease suggests that certain microbial taxa may be sensitive to high-temperature treatments, potentially hindering DNA recovery. Importantly, the authors cautioned that HotSHOT may reduce sensitivity for detecting low-abundance bacteria, such as *Phytoplasma*, which are often represented by only a few reads in 16S rRNA amplicon datasets. These results highlight the importance of using targeted detection methods when surveying rare or low-titer microbes and emphasize potential biases introduced by both high-temperature pretreatments and amplicon sequencing. Overall, this study reinforces the need for careful optimization of extraction protocols, particularly when profiling diverse microbial communities.

Future studies should investigate whether active PK affects DNA quality during extended storage or repeated freeze–thaw cycles, particularly for applications requiring high-quality DNA or long-term sample preservation.

While PK is commonly used solely to increase DNA yield, soaking specimens in PK has also been demonstrated to be an effective method for clearing soft tissue from arthropods. After this treatment, only the exoskeleton remains, allowing for clear visualization of the genitalia morphology, which is useful for species identification for some insect groups including leafhoppers. The use of PK to prepare genitalia capsules for microscopic examination has been shown to be more effective than the commonly used KOH (Martinelli et al. 2017). PK has been observed to degrade the exoskeleton to a lesser extent than KOH, retaining more defined features such as texture and sclerotization patterns. This makes PK a viable and potentially preferable method for clearing soft tissue. The inclusion of PK in DNA extractions is particularly well suited for insect groups for which genitalic morphology is a key diagnostic feature. Our refined protocol included additional abrasive steps, including bleaching, vortexing, and a 24-h incubation in a 55 °C water bath with TES buffer, yet no damage to the genital structures was observed. Coloration and external patterning on the specimens’ surfaces were slightly lightened after extraction; however, the main patterns remained stable. Since coloration is not a diagnostic character for the insect group used in our study, this fading was not a concern. However, before applying our protocol to insect groups for which coloration is a key identifying trait, we recommend conducting preliminary tests to evaluate any potential changes, especially when working with precious voucher specimens.

When evaluating the retention of morphological characters in preserved specimens, it is also important to consider potential issues associated with using 95% ethanol (EtOH) as a preservation method. While we recommend 95% EtOH due to its effectiveness in preserving DNA (King and Porter 2004, Nagy 2010), its high concentration can cause brittleness in specimen exoskeletons, often resulting in the loss of limbs. This effect is especially pronounced in insects with delicate or thin exoskeletons (Marquina et al. 2021). We encourage researchers to take this into account when working with fragile specimens, particularly those for which limb morphology is important for identification. In line with previous recommendations (eg Brooks 1993), our study promotes the routine preservation and cataloging of voucher specimens for all species examined, even when the primary focus is on associated microbiomes. By integrating this practice into our workflow, we aim to strengthen the documentation of host–microbe associations and encourage its broader adoption in pathogen surveillance and diagnostic studies.

Currently, there is no consensus on the optimal bleach concentration and exposure duration that guarantees elimination of contaminant DNA of the external surface of the insect while also not compromising the integrity of the insect and gut content DNA (Korlević and Meyer 2019, Koehn et al. 2020, Oh et al. 2020, de Silva Wijeyeratne and Gweon 2025). We soaked insects in a 2.5% bleach solution for 5 min to degrade potential contaminant DNA while still ensuring that internal DNA would remain undisturbed. Our findings showed that bleaching neither decreased the total DNA yield, nor compromised the quantification of associated low-titer microbial cells. However, since our goal was to test the effects of bleaching on downstream analysis, we did not confirm the presence of contaminant DNA on insects prior to bleaching nor did we test DNA samples for contaminant DNA. Because of this, we cannot guarantee that our protocol will remove all external contaminant DNA on insects that undergo this treatment. We advise others using our protocol to take this into account and make an informed decision on the ideal concentration and duration of bleach treatment for their taxa and study purposes. Previous studies have shown the effectiveness of bleaching in reducing surface contamination without negatively impacting the integrity of target DNA. For instance, Greenstone et al. (2012) demonstrated that bleaching insect samples prior to DNA extraction enhanced the purity of microbial DNA, particularly for gut microbiome studies. Huszarik et al. (2023) also confirmed that bleaching is a reliable method for minimizing contaminants while preserving DNA yield and amplification effectiveness. They also found that gut content DNA was not different between specimens that were bleached and those that were not, indicating that external bleaching of specimens does not impact internal DNA, though this could become a concern if bleach entered the gut. Despite the proven effectiveness of bleaching in eliminating external contaminant DNA, a critical concern is whether the bleaching process may degrade the specimen’s own DNA (Kemp and Smith 2005) and therefore reduce the final yield or interfere with downstream analyses.

### Nondestructive Extraction and Vouchering

Insect specimens preserved in museum collections potentially offer a wealth of genetic information on difficult to obtain species, which can enhance our understanding of many facets of biology, but such specimens often represent species that are rare and difficult to collect and/or localities that are difficult to access, so museum curators may be reluctant to allow destructive DNA sampling from specimens under their care. For these reasons, it is important to develop nondestructive DNA extraction methods which produce high yield and quality of DNA but leave the specimens themselves intact. In addition to our findings, others have also implemented a variety of strategies to extract DNA from museum specimens while keeping the insect bodies intact. Patzold et al. (2020) extracted DNA from museum specimens of lepidopteran type specimens that were 20 to 214 yr old using a minimally destructive method which utilized one whole leg from each specimen. Using this method, they retrieved an average DNA concentration of 12.1 ng/μl, a suitable amount to perform qPCR analyses. Mullin et al. (2023) used a similar approach in which they used a single leg from bumblebee specimens that were up to 113 yr old with the goal of obtaining genome-wide data to serve as a baseline when investigating population changes at a genetic level. DNA was successfully extracted using this method, although there were high levels of fragmentation with most DNA fragments containing less than 100 base pairs. Mullin et al. suggested that DNA degradation in specimens increases with time since death, which may cause issues when trying to amplify DNA from older specimens. Thomsen et al. (2009) extracted DNA from whole-body beetle museum specimens, including some from 1820 AD. They successfully extracted and amplified DNA for specimens of all ages.

While this study focused on a single insect species, we believe the protocol can be successfully applied to other taxa. For larger species, we recommend adjusting the volume of TES buffer to fully cover the insect body in the tube, as well as proportionally increasing the amounts of PK and chelating resin. However, we cannot guarantee the effectiveness of this protocol for taxa with different microbiome compositions. Therefore, we recommend testing this protocol on a case-by-case basis to verify its suitability for different host–microbe associations.

The main goal of this study was to provide an improved, nondestructive, fast, inexpensive, nontoxic chelating resin-based DNA extraction protocol intended for application on insect museum vouchers and their associated microbiomes. Achieving a high DNA yield is critical for studies that require multiple downstream analyses, such as DNA sequencing or metagenomic studies, and for ensuring reproducibility in experiments. The silica column-based Qiagen kit that served here as an internal reference is known to be a reliable method for extracting high-quality DNA from insect specimens (Patzold et al. 2020). The inclusion of samples processed using silica columns allowed us to ensure that samples treated similarly, differing only in the use of chelating resin, could still yield comparable results. Consistent with prior studies that employ both destructive (Lienhard and Schäffer 2019) and minimally destructive (Casquet et al. 2012,) methods, samples purified with chelating resin consistently yielded higher quantities of DNA compared to those purified using silica columns, which may be due to our replacement of the custom lysis buffer with TES. Using a nondestructive approach, we also obtained a significantly higher amount of phytoplasma DNA when using chelating resin, along with longer sequences of the phytoplasma 16S barcode gene. Other studies have shown that samples collected using nondestructive methods can be successfully metabarcoded (Carew et al. 2018, Martoni et al. 2022). Our results indicate that the chelating resin-based protocol is suitable for retrieving an acceptable amount of total DNA, even though the quality is slightly inferior. Overall, when comparing DNA quality between treatments and for each protocol separately, comparable results were retrieved. This emphasizes the viability of such nondestructive methods for studies of the microbiomes of museum specimens. However, it is important to note that nondestructive methods may not guarantee homogenous tissue lysis which may impact DNA integrity, though we did not observe this issue and were able to successfully amplify and sequence our samples.

Based on our findings and those of previous studies, for gut-associate microbiome studies we recommend a protocol that includes a prelysis bleaching step, uses PK without a postlysis heat inactivation step, and utilizes a resin-based purification method. The final protocol can be viewed at 10.17504/protocols.io.bp2l6x54rlqev1. This protocol is suited for the nondestructive extraction of DNA from museum arthropod specimens, particularly for extractions targeting gut content and microbial genome and for specimens representing species for which identification requires the preservation of genitalic morphology.

## Supplementary Material

ieaf062_suppl_Supplementary_Tables_S1-S5_Figures_S1-S4
