# Supplementary material for: A non-destructive, fast, inexpensive, non-toxic chelating resin-based DNA extraction protocol for insect voucher specimens and associated microbiomes
Source: J Insect Sci. 2025 Jun 3;25(3):17. doi: 10.1093/jisesa/ieaf062 (PMC12132034; doi:10.1093/jisesa/ieaf062)

SUPPLEMENTARY MATERIALS

Table S1. Modified Qiagen DNeasy® Blood & Tissue Kit protocol.

| 1 | Cell lysis according to steps 1-6 of Chelex protocol optimized in this paper  (dx.doi.org/10.17504/protocols.io.bp2l6x54rlqe/v1) |
| --- | --- |
| 2 | Add 200 µL Buffer AL. Vortex and incubate samples at 55 °C for 10 min. |
| 3 | Add 200 μl ethanol (96–100%). Vortex. |
| 4 | Pipette mixture into a DNeasy Mini spin column in a 2 mL collection tube. Centrifuge at 8,000 rpm for 1 min and discard flow-through. |
| 5 | Add 500 µL Buffer AW1. Centrifuge at 8,000 rpm for 1 min and discard flow-through. |
| 6 | Add 500 µL Buffer AW2. Centrifuge at 14,000 rpm for 3 min and discard flow-through. |
| 7 | Move the spin column to a new microcentrifuge tube. |
| 8 | Elute DNA from the spin column by adding 50 µL Buffer AE. Incubate for 3 min at 70 °C and centrifuge for 1-2 min at 8,000 rpm. |
| 9 | Store DNA at -20° C. |

Table S2. Average starting quantity (SQ), mean Cq values and standard deviations for standard curves and non-template controls (NTCs) across the five qPCR run used to quantify phytoplasma titer in insect samples.

| ***Standard*** | ***Fluorescent dye*** | ***Mean SQ*** | ***Mean Cq ± SD*** |
| --- | --- | --- | --- |
| Phy 7 | FAM | 38,800,000 | 15.33 ± 0.09 |
| Phy 5 | FAM | 388,000 | 22.44 ± 0.36 |
| Phy 3 | FAM | 3,880 | 29.88 ± 0.30 |
| Phy 2 | FAM | 388 | 33.87 ± 1.30 |
| Phy 1 | FAM | 38.8 | 36.94 ± 1.09 |
| NTC | FAM | 0 | 38.51 ± 0.07 |
| Au 5 | HEX | 11 | 20.16 ± 0.10 |
| Au 4 | HEX | 1.1 | 24.81 ± 0.56 |
| Au 3 | HEX | 0.11 | 29.19 ± 0.35 |
| Au 2 | HEX | 0.011 | 32.83 ± 0.44 |
| Au 1 | HEX | 0.00110 | 36.83 ± 0.65 |
| NTC | HEX | 0 | 46.39 ± 0.00 |

Table S3. Data of quantification of samples used to compare DNA yield and absolute quantification of phytoplasma cells between treatments in pre- (bleaching) and post-lysis (PK inactivation) for two different extraction protocols (bead-based and silica column-based). The yield (ng/sample) and phytoplasma quantification (phytoplasma unit/ng insect DNA) were calculated for each paired sample. The average Cq values and the standard deviation for the two technical replicates are also reported. Samples highlighted in red represent either insects not exposed to phytoplasma (C) or non template control (NTC) used as blacks in qPCR plates.

| ***Bleach*** | ***Proteinase K inactivation*** | ***Protocol*** | ***Individual*** | ***Yield*** | ***Absolute phytoplasma quantification*** | ***Phytoplasma average Cq (± SD)*** | ***Housekeeping average Cq (± SD)*** |
| --- | --- | --- | --- | --- | --- | --- | --- |
| yes | yes | chelex | C1 | 268.8 | 0.00E+00 | 39.97 ± 0.44 | 26.00 ± 0.01 |
| yes | yes | chelex | 2 | 807 | 2.21E+05 | 24.80 ± 0.04 | 26.59 ± 0.04 |
| yes | yes | chelex | 3 | 777 | 6.27E+04 | 30.14 ± 0.03 | 30.59 ± 0.00 |
| yes | yes | chelex | 4 | 1224 | 2.59E+05 | 26.34 ± 0.08 | 28.65 ± 0.09 |
| yes | yes | chelex | 5 | 296.4 | 9.30E+05 | 26.01 ± 0.08 | 35.07 ± 0.27 |
| yes | yes | chelex | 6 | 300 | 8.62E+04 | 30.12 ± 0.06 | 31.13 ± 0.05 |
| yes | yes | chelex | C | 92.4 | 0.00E+00 | 42.18 ± 0.00 | 26.17 ± 0.11 |
| yes | yes | chelex | 8 | 30.3 | 4.37E+05 | 21.48 ± 0.01 | 23.93 ± 0.01 |
| yes | yes | chelex | 9 | 13.68 | 2.34E+06 | 23.52 ± 0.11 | 29.21 ± 0.02 |
| yes | yes | chelex | 10 | 33.3 | 1.22E+06 | 20.21 ± 0.06 | 24.25 ± 0.11 |
| yes | yes | qiagen | C1 | 27.4 | 0.00E+00 | 38.45 ± 0.08 | 24.15 ± 0.09 |
| yes | yes | qiagen | 2 | 25.5 | 3.24E+05 | 22.66 ± 0.04 | 24.78 ± 0.05 |
| yes | yes | qiagen | 3 | 4.36 | 1.23E+05 | 27.14 ± 0.05 | 28.29 ± 0.14 |
| yes | yes | qiagen | 4 | 31.6 | 1.64E+05 | 23.91 ± 0.14 | 25.04 ± 0.02 |
| yes | yes | qiagen | 5 | 0 | 7.23E+05 | 23.95 ± 0.04 | 32.34 ± 0.00 |
| yes | yes | qiagen | 6 | 10.2 | 1.41E+05 | 28.09 ± 0.90 | 29.79 ± 1.52 |
| yes | yes | qiagen | C | 15.6 | 0.00E+00 | >50 ± 0.00 | 25.55 ± 0.04 |
| yes | yes | qiagen | 8 | 15.4 | 4.51E+05 | 23.78 ± 0.07 | 26.65 ± 0.09 |
| yes | yes | qiagen | 9 | 13.1 | 1.55E+06 | 22.30 ± 0.06 | 27.08 ± 0.07 |
| yes | yes | qiagen | 10 | 14.1 | 1.49E+06 | 22.13 ± 0.03 | 26.82 ± 0.03 |
| yes | no | chelex | 11 | 1476 | 9.92E+04 | 28.37 ± 0.05 | 29.34 ± 0.02 |
| yes | no | chelex | 12 | 822 | 1.20E+05 | 28.19 ± 0.06 | 29.47 ± 0.03 |
| yes | no | chelex | 13 | 1011 | 1.56E+05 | 27.95 ± 0.15 | 29.63 ± 0.11 |
| yes | no | chelex | 14 | 558 | 8.19E+04 | 28.61 ± 0.15 | 29.29 ± 0.17 |
| yes | no | chelex | 15 | 738 | 9.87E+05 | 25.21 ± 0.28 | 29.68 ± 0.49 |
| yes | no | chelex | 16 | 387 | 5.41E+05 | 25.59 ± 0.10 | 29.07 ± 0.07 |
| yes | no | chelex | 17 | 190.8 | 1.66E+05 | 26.45 ± 0.11 | 28.01 ± 0.07 |
| yes | no | chelex | 18 | 1212 | 2.83E+06 | 24.47 ± 0.02 | 30.64 ± 0.10 |
| yes | no | chelex | 19 | 606 | 1.95E+06 | 24.96 ± 0.28 | 30.57 ± 0.32 |
| yes | no | chelex | 20 | 468 | 7.98E+05 | 25.73 ± 0.17 | 28.61 ± 0.09 |
| yes | no | chelex | C | 381 | 0.00E+00 | 39.90 ± 0.00 | 29.23 ± 0.02 |
| yes | no | qiagen | 11 | 176 | 1.22E+05 | 27.20 ± 0.04 | 28.34 ± 0.05 |
| yes | no | qiagen | 12 | 110 | 1.55E+05 | 27.17 ± 0.14 | 28.73 ± 0.24 |
| yes | no | qiagen | 13 | 17.2 | 8.11E+04 | 27.33 ± 0.00 | 27.78 ± 0.04 |
| yes | no | qiagen | 14 | 21.1 | 3.73E+04 | 28.41 ± 0.12 | 27.68 ± 0.08 |
| yes | no | qiagen | 15 | 25.6 | 1.24E+06 | 23.11 ± 0.05 | 27.63 ± 0.07 |
| yes | no | qiagen | 16 | 31.1 | 4.22E+05 | 26.41 ± 0.01 | 29.59 ± 0.05 |
| yes | no | qiagen | 17 | 20.4 | 1.79E+05 | 26.87 ± 0.21 | 28.62 ± 0.18 |
| yes | no | qiagen | 18 | 56 | 1.97E+06 | 24.63 ± 0.11 | 30.20 ± 0.10 |
| yes | no | qiagen | 19 | 19.1 | 8.15E+05 | 24.36 ± 0.05 | 28.35 ± 0.04 |
| yes | no | qiagen | 20 | 25.5 | 1.06E+06 | 24.41 ± 0.03 | 27.73 ± 0.05 |
| yes | no | qiagen | C | 20.3 | 0.00E+00 | >50 ± 0.00 | 26.99 ± 0.08 |
|  |  | Plate1 |  |  |  | 38.58 ± 0.66 | 46.39 ± 0.00 |
| no | yes | chelex | 21 | 759 | 4.19E+05 | 24.73 ± 1.74 | 27.80 ± 1.79 |
| no | yes | chelex | 22 | 1212 | 1.06E+05 | 30.06 ± 1.51 | 31.28 ± 1.70 |
| no | yes | chelex | 23 | 591 | 7.57E+05 | 26.81 ± 0.06 | 35.59 ± 0.06 |
| no | yes | chelex | 24 | 714 | 3.69E+05 | 27.28 ± 0.04 | 34.88 ± 0.20 |
| no | yes | chelex | 25 | 444 | 1.37E+05 | 29.66 ± 2.10 | 31.16 ± 2.04 |
| no | yes | chelex | 26 | 828 | 6.53E+05 | 25.60 ± 1.27 | 29.52 ± 1.33 |
| no | yes | chelex | 27 | 459 | 2.59E+04 | 32.97 ± 1.19 | 32.19 ± 1.71 |
| no | yes | chelex | 28 | 594 | 5.34E+05 | 27.90 ± 1.97 | 31.61 ± 1.99 |
| no | yes | chelex | 29 | 759 | 5.65E+05 | 27.03 ± 2.17 | 30.86 ± 2.39 |
| no | yes | chelex | 30 | 113.1 | 2.69E+06 | 23.40 ± 1.52 | 29.55 ± 1.59 |
| no | yes | chelex | C | 114.3 | 0.00E+00 | 38.22 ± 0.00 | 33.99 ± 0.02 |
| no | yes | qiagen | 21 | 22.9 | 2.53E+05 | 23.41 ± 1.87 | 25.63 ± 2.16 |
| no | yes | qiagen | 22 | 10.4 | 9.47E+04 | 26.15 ± 1.56 | 26.88 ± 1.79 |
| no | yes | qiagen | 23 | 3.92 | 4.24E+05 | 25.22 ± 0.05 | 32.84 ± 0.22 |
| no | yes | qiagen | 24 | 5.04 | 1.44E+05 | 26.75 ± 0.04 | 32.66 ± 0.12 |
| no | yes | qiagen | 25 | 5.6 | 5.61E+04 | 26.25 ± 1.33 | 26.10 ± 1.52 |
| no | yes | qiagen | 26 | 14.1 | 3.32E+05 | 21.16 ± 1.52 | 23.64 ± 1.77 |
| no | yes | qiagen | 27 | 9.32 | 3.40E+03 | 29.60 ± 0.48 | 24.97 ± 0.82 |
| no | yes | qiagen | 28 | 4.64 | 1.73E+05 | 25.47 ± 2.53 | 27.27 ± 2.97 |
| no | yes | qiagen | 29 | 15.4 | 1.82E+05 | 22.75 ± 0.97 | 24.33 ± 1.21 |
| no | yes | qiagen | 30 | 5 | 2.33E+06 | 20.62 ± 0.66 | 26.29 ± 0.58 |
| no | yes | qiagen | C | 3 | 0.00E+00 | >50 ± 0.00 | 31.57 ± 0.02 |
| no | no | chelex | 31 | 1464 | 2.88E+05 | 22.61 ± 0.96 | 24.93 ± 1.14 |
| no | no | chelex | 32 | 1236 | 1.41E+06 | 25.26 ± 0.53 | 30.48 ± 0.53 |
| no | no | chelex | 33 | 930 | 9.78E+04 | 29.93 ± 1.77 | 30.88 ± 1.66 |
| no | no | chelex | 34 | 606 | 6.00E+05 | 26.67 ± 1.91 | 30.67 ± 2.31 |
| no | no | chelex | 35 | 576 | 1.39E+05 | 28.91 ± 0.13 | 30.19 ± 0.06 |
| no | no | chelex | 36 | 717 | 1.36E+04 | 32.33 ± 0.28 | 29.79 ± 0.03 |
| no | no | chelex | 37 | 2916 | 1.02E+05 | 35.00 ± 0.19 | 36.52 ± 0.29 |
| no | no | chelex | 38 | 774 | 3.29E+05 | 26.40 ± 0.03 | 28.92 ± 0.06 |
| no | no | chelex | 39 | 369 | 7.75E+04 | 31.05 ± 0.10 | 31.54 ± 0.05 |
| no | no | chelex | 40 | 260.4 | 2.84E+06 | 25.10 ± 0.01 | 35.99 ± 0.06 |
| no | no | chelex | C | 264 | 4.06E+01 | 39.69 ± 0.04 | 27.52 ± 0.05 |
| no | no | qiagen | 31 | 12.4 | 1.57E+06 | 22.56 ± 1.22 | 27.73 ± 1.29 |
| no | no | qiagen | 32 | 96.4 | 2.35E+05 | 25.36 ± 2.46 | 27.57 ± 2.72 |
| no | no | qiagen | 33 | 13 | 1.95E+04 | 28.29 ± 2.48 | 26.62 ± 2.95 |
| no | no | qiagen | 34 | 5.76 | 6.41E+04 | 26.49 ± 1.02 | 26.58 ± 1.18 |
| no | no | qiagen | 35 | 14.6 | 7.14E+04 | 26.33 ± 0.06 | 26.06 ± 0.07 |
| no | no | qiagen | 36 | 71.2 | 7.93E+03 | 30.07 ± 0.01 | 26.27 ± 0.03 |
| no | no | qiagen | 37 | 22.7 | 6.05E+05 | 24.58 ± 0.07 | 27.97 ± 0.05 |
| no | no | qiagen | 38 | 15.6 | 1.09E+05 | 27.13 ± 0.54 | 27.71 ± 0.48 |
| no | no | qiagen | 39 | 8.72 | 2.75E+04 | 29.21 ± 0.07 | 27.56 ± 0.02 |
| no | no | qiagen | 40 | 6.84 | 1.28E+06 | 25.61 ± 0.03 | 35.17 ± 0.03 |
| no | no | qiagen | C | 20.6 | 0.00E+00 | 37.43 ± 0.00 | 27.96 ± 0.10 |
| - | - | Plate2 | - | - | - | >50± 0.0 | >50± 0.0 |


Table S4. Paired contrast of the results from the post-hoc Tukey test conducted on the linear mixed-effects model assessing the effect of bleaching (yes or no), proteinase K inactivation (yes or no), and protocol (chelex or qiagen).

| ***Contrast*** | ***Estimate*** | ***SE*** | ***df*** | ***t.ratio*** | ***p.value*** |
| --- | --- | --- | --- | --- | --- |
| noB noPKin CH - B noPkin CH | 0.196 | 0.398 | 36 | 0.492 | 1.000 |
| noB noPKin CH - noB PKin CH | 0.330 | 0.398 | 36 | 0.829 | 0.990 |
| noB noPKin CH - B Pkin CH | 1.504 | 0.398 | 36 | 3.775 | 0.012 |
| noB noPKin CH - noB noPKin Q | 3.764 | 0.356 | 36 | 10.570 | 3.775E-11 |
| noB noPKin CH - B noPkin Q | 3.068 | 0.398 | 36 | 7.703 | 1.076E-07 |
| noB noPKin CH - noB PKin Q | 4.445 | 0.398 | 36 | 11.159 | 8.201E-12 |
| noB noPKin CH - B Pkin Q | 4.153 | 0.398 | 36 | 10.427 | 5.488E-11 |
| B noPkin CH - noB PKin CH | 0.134 | 0.398 | 36 | 0.337 | 1.000 |
| B noPkin CH - B Pkin CH | 1.308 | 0.398 | 36 | 3.283 | 0.043 |
| B noPkin CH - noB noPKin Q | 3.568 | 0.398 | 36 | 8.958 | 2.931E-09 |
| B noPkin CH - B noPkin Q | 2.872 | 0.356 | 36 | 8.065 | 3.738E-08 |
| B noPkin CH - noB PKin Q | 4.249 | 0.398 | 36 | 10.667 | 2.934E-11 |
| B noPkin CH - B Pkin Q | 3.957 | 0.398 | 36 | 9.934 | 2.022E-10 |
| noB PKin CH - B Pkin CH | 1.173 | 0.398 | 36 | 2.946 | 0.093 |
| noB PKin CH - noB noPKin Q | 3.434 | 0.398 | 36 | 8.621 | 7.572E-09 |
| noB PKin CH - B noPkin Q | 2.738 | 0.398 | 36 | 6.875 | 1.269E-06 |
| noB PKin CH - noB PKin Q | 4.114 | 0.356 | 36 | 11.554 | 2.916E-12 |
| noB PKin CH - B Pkin Q | 3.823 | 0.398 | 36 | 9.598 | 5.015E-10 |
| B Pkin CH - noB noPKin Q | 2.260 | 0.398 | 36 | 5.675 | 4.806E-05 |
| B Pkin CH - B noPkin Q | 1.565 | 0.398 | 36 | 3.928 | 0.008 |
| B Pkin CH - noB PKin Q | 2.941 | 0.398 | 36 | 7.384 | 2.768E-07 |
| B Pkin CH - B Pkin Q | 2.649 | 0.356 | 36 | 7.440 | 2.346E-07 |
| noB noPKin Q - B noPkin Q | -0.696 | 0.398 | 36 | -1.747 | 0.658 |
| noB noPKin Q - noB PKin Q | 0.681 | 0.398 | 36 | 1.709 | 0.682 |
| noB noPKin Q - B Pkin Q | 0.389 | 0.398 | 36 | 0.976 | 0.975 |
| B noPkin Q - noB PKin Q | 1.376 | 0.398 | 36 | 3.455 | 0.028 |
| B noPkin Q - B Pkin Q | 1.085 | 0.398 | 36 | 2.723 | 0.148 |
| noB PKin Q - B Pkin Q | -0.292 | 0.398 | 36 | -0.732 | 0.995 |

Table S5. NanoDrop results from a subset of samples.

| ***Bleach*** | ***Proteinase K inactivation*** | ***Protocol*** | ***ng/uL DNA*** | ***260/280*** | ***260/230*** |
| --- | --- | --- | --- | --- | --- |
| yes | yes | qiagen | 79.2 | 1.94 | 2.41 |
| yes | yes | qiagen | 80.5 | 1.94 | 2.26 |
| no | no | qiagen | 78.5 | 1.96 | 1.41 |
| no | no | qiagen | 73 | 1.94 | 1.52 |
| no | yes | qiagen | 84.7 | 1.88 | 2.14 |
| no | yes | qiagen | 106.6 | 2.05 | 1.48 |
| yes | no | qiagen | 100.3 | 2.06 | 2.2 |
| yes | no | qiagen | 71 | 2.14 | 2.04 |
| yes | yes | chelex | 203.9 | 0.92 | 0.34 |
| yes | yes | chelex | 168.5 | 1.05 | 0.32 |
| yes | yes | chelex | 174.9 | 0.98 | 0.76 |
| yes | yes | chelex | 139 | 1.18 | 0.83 |
| no | yes | chelex | 145.2 | 1.14 | 1.01 |
| no | yes | chelex | 165.6 | 1.25 | 0.92 |
| yes | no | chelex | 148 | 1.13 | 0.93 |
| yes | no | chelex | 121.6 | 1.1 | 0.82 |
| no | no | chelex | 135.7 | 1.02 | 0.65 |
| no | no | chelex | 172.2 | 1.17 | 0.76 |
| no | no | chelex | 249.5 | 0.99 | 0.7 |

Figure S1. Results of post-hoc Tukey test of the linear-mixed effects model comparing the DNA yield (ng/sample) of treatments varying in bleach (yes or no), proteinase K inactivation (yes or no), and protocol (chelex or qiagen).


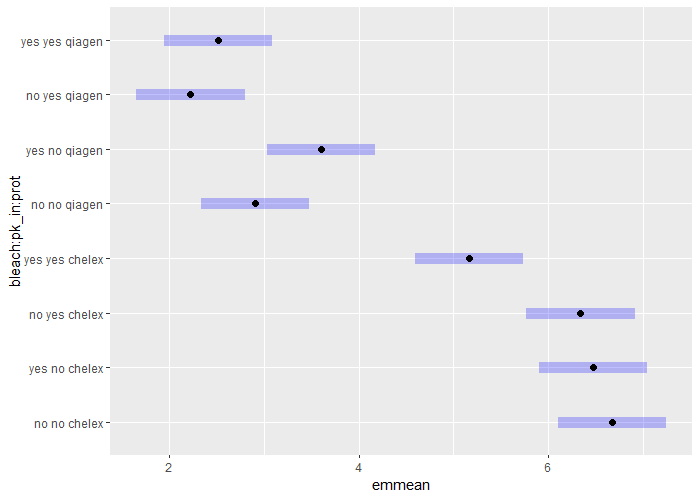


Figure S2. Agarose gel electrophoresis of 10 DNA samples extracted from five biological replicates from the treatment Bleaching + no PK inactivation and for each DNA aliquot extracted using Chelex or Qiagen protocol. Each well was loaded with 100 ng of total DNA per sample. Lengths in kilobase pairs are shown on the left-hand side as specified by the 1 Kb ladder used.


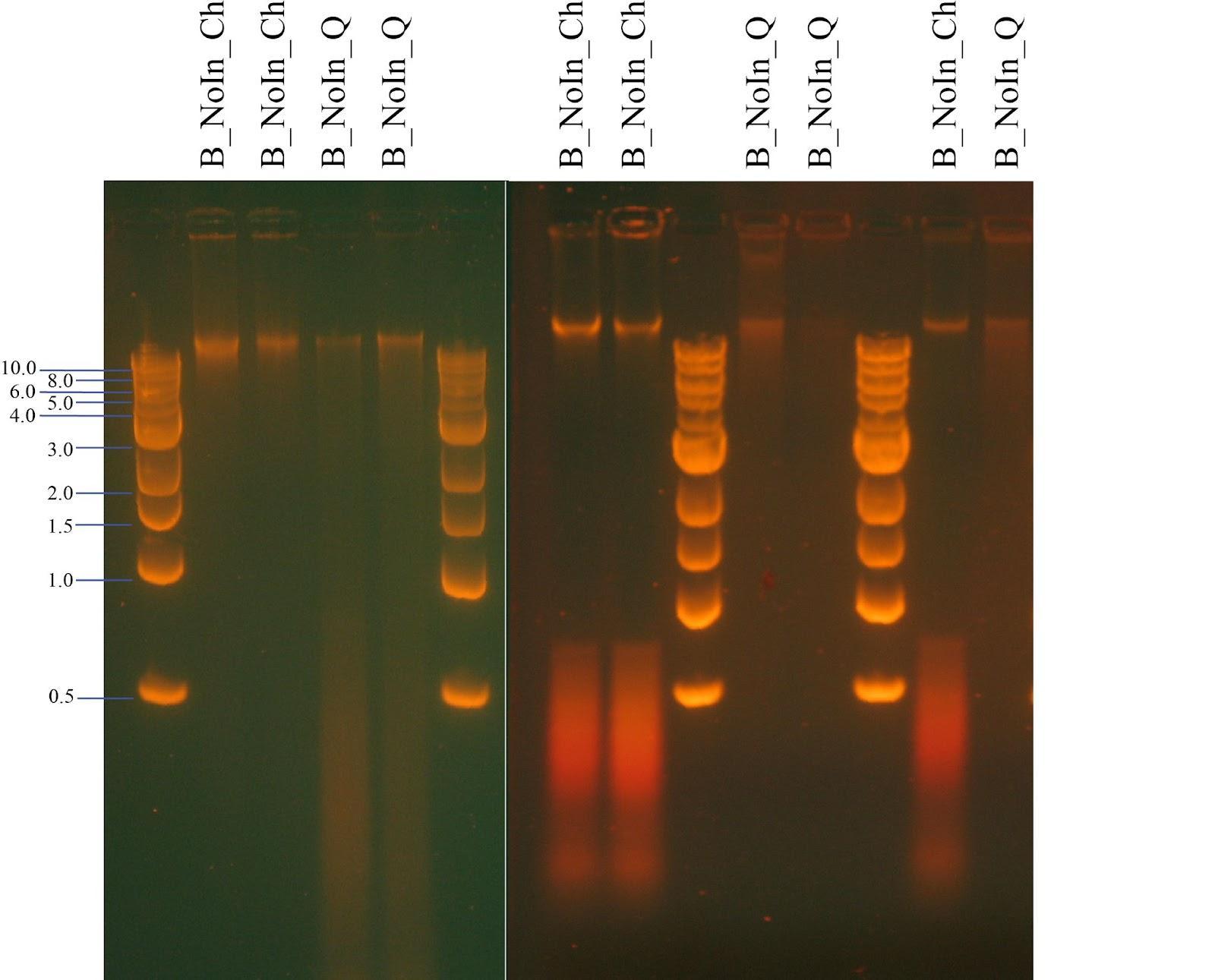


Figure S3. Photographs of the aedeagus (a, b) and subgenital plates (c, d) of genitalia dissected from an individual that did not undergo extraction (a, c) and an individual that underwent the most intensive extraction treatment (Bleaching + Proteinase K inactivation) (b, d). Illinois Natural History Survey Insect Collection accession codes are included below the associated individuals. Photographs were taken using a Jenoptik Gryphax Arktur microscope camera (Jenoptik Optical Systems GmbH, Jena, Germany) mounted to an Olympus BX41 microscope (Olympus Corp. Tokyo, Japan). Photographs at different focal points were combined using Jenoptik Gryphax software (Jenoptik Optical Systems GmbH, Jena, Germany).


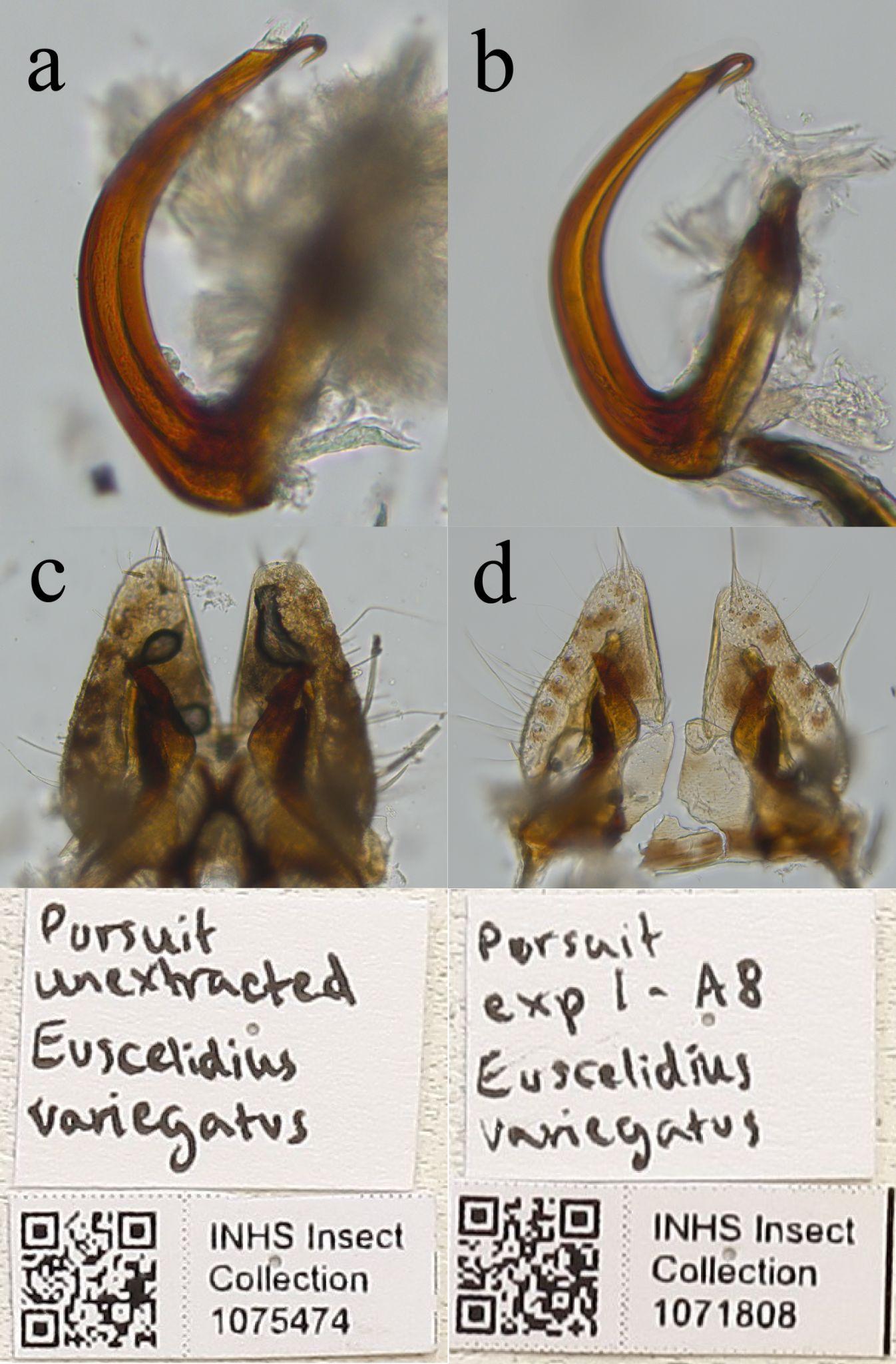


Figure S4. Photographs of dorsal (a-f) and ventral (g-k) aspects of Euscelidius variegatus individuals, including one museum specimen that has not undergone extraction and was preserved without EtOH (a, image courtesy of Michelle Kohler), one individual that has not undergone extraction and was preserved in 95% EtOH (b & g) and four individuals that did undergo extraction with all combinations of extraction treatments represented: (1) Bleaching + Proteinase K inactivation (c & h); (2) Bleaching + No proteinase K inactivation (d & i); (3) No bleaching + Proteinase K inactivation (e & j); and (4) No bleaching + No proteinase K inactivation (f & k). The included 1 mm scale bar is to scale with figures b-f. Illinois Natural History Survey Insect Collection accession codes are included below the associated individual. Photographs were taken by the first author using a Canon DX1 SLR camera (Canon Inc., Tokyo, Japan) with a Canon MP-E 65mm macro lens (Canon Inc., Tokyo, Japan) mounted to a motorized lift. Photographs were taken at multiple focal planes then combined using Zerene Stacker (Zerene Systems, Richland, WA, USA).


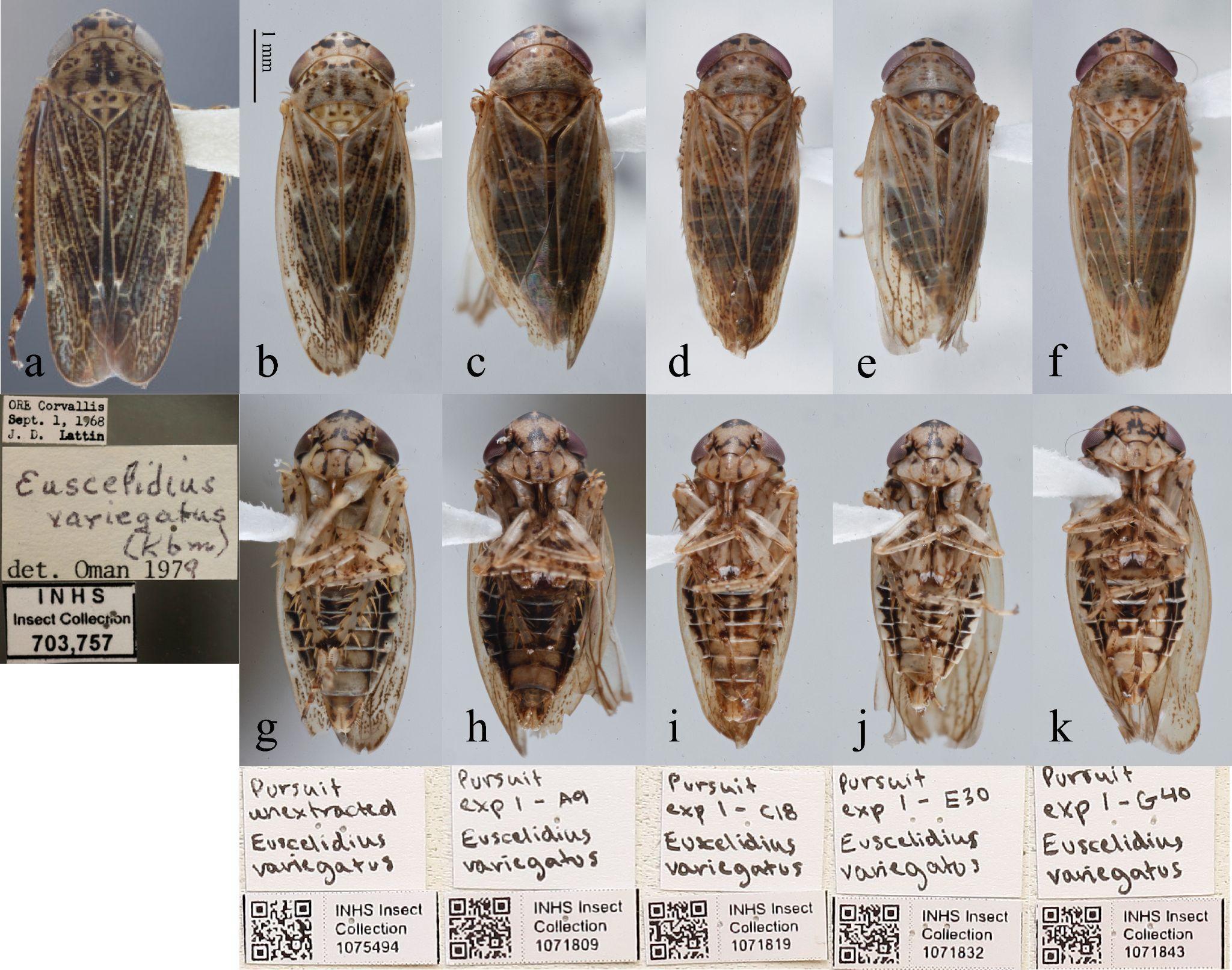

Supplement: ieaf062_suppl_Supplementary_Tables_S1-S5_Figures_S1-S4 [file ieaf062_suppl_supplementary_tables_s1-s5_figures_s1-s4.docx]
